# Synergistic Interactions Between the Quinolone-Derived Compound HT61 and Tobramycin Against a Range of Clinical Isolates of *Pseudomonas aeruginosa* In Vitro

**DOI:** 10.1007/s00408-025-00855-x

**Published:** 2025-11-27

**Authors:** B. Ramsden, A. V. Kelis, M.-E. Faure, C. P. Page, R. T. Amison

**Affiliations:** Unit of Pulmonary Pharmacology, Institute of Pharmaceutical Science, School of Cancer and Pharmaceutical Science, King’s College London, Waterloo Campus, 5.72 Franklin-Wilkins Building, London, SE1 9NH UK

**Keywords:** Respiratory infections, Antimicrobial resistance, Combination therapies, Synergy, *P. aeruginosa*

## Abstract

**Background:**

Antimicrobial-resistant pathogens such as *Pseudomonas aeruginosa (P. aeruginosa)* represent a significant challenge to patients with respiratory diseases including Cystic Fibrosis (CF) and chronic obstructive pulmonary disease (COPD), where treatment of such infections is exacerbated by a shortage of new and effective antibiotic classes. One novel approach utilises ‘antibiotic enhancers’ that potentiate the antimicrobial activity of existing antibiotics. HT61, a small quinolone-derived compound, potentiates the activity of the aminoglycosides tobramycin and gentamicin against *Staphylococcus aureus* and *P. aeruginosa.* In this study, we have investigated synergism between tobramycin and HT61 using a panel of tobramycin-sensitive and -resistant clinical isolates of *P. aeruginosa* from CF patients.

**Methods:**

Microdilution methods and chequerboard analysis were used to evaluate antimicrobial synergy of drug combinations against 63 isolates. Bacterial time-kill assays and biofilm eradication assays were then used to further characterise antimicrobial synergy against 13 selected isolates.

**Results:**

74% of isolates (47/63) demonstrated evidence of either positive interactions (29/63) determined by a Fractional Inhibitory Concentration Index (FICI) ≤ 1.0, or synergy (18/63) determined by an FICI value of ≤ 0.5. Using a sub-selection of these isolates, clear augmentation of tobramycin’s antimicrobial activity was observed in both time-kill assays and biofilm eradication assays regardless of FICI classification with significant reductions observed in combination therapies vs monotherapies.

**Conclusions:**

The expansion of previous studies highlighting the potentiating capabilities of HT61 on/with antibiotics in vivo across a further 63 clinical isolates of *P. aeruginosa* in a laboratory setting further highlights the potential therapeutic benefits of HT61-tobramycin combinations in respiratory diseases associated with drug-resistant *P. aeruginosa*.

## Introduction

*Pseudomonas aeruginosa* (*P. aerguinosa*) is a common opportunistic Gram-negative pathogen implicated in both acute and chronic infections in immunocompromised and hospitalised patients. Two of the most clinically challenging situations include persistent chronic lung infections and acute infective pulmonary exacerbations (PEx) in patients with Cystic Fibrosis (CF), Chronic Obstructive Pulmonary Disease (COPD) and non-CF bronchiectasis [1–5]. One particular challenge associated with chronic *P. aeruginosa* infections, is the development into biofilms providing resistance against both host immune responses and antimicrobial therapy. Additionally, the continued emergence of intrinsic and acquired resistance mechanisms has led to reduced antimicrobial activity against *P. aeruginosa* [6–9], with the prevalence of multidrug-resistant (MDR) and extensively resistant (XDR) *P. aeruginosa* reaching between 15 and 30% in some geographical areas [10–12].

Tobramycin remains the standard of care treatment for *P. aeruginosa* infections, with chronic infections utilising inhaled tobramycin (either as a solution or dry powder) [2] and for acute PEx, intravenous tobramycin. However, due to the increased prevalence of tobramycin-resistant *P. aeruginosa* strains and a paucity of new classes of antibiotics in development, these infections are becoming increasingly difficult to treat, requiring ever higher doses of antibiotic associated with systemic toxicity such as both oto- and nephro-toxicity. Therefore, novel strategies that either refresh or extend the impact of existing antibiotics are required. Current approaches have evaluated: structural optimization of antibiotics to overcome bacterial resistance mechanisms; combinations of multiple antimicrobial compounds; and combinations of antimicrobials with compounds without direct antimicrobial activity termed ‘antibiotic enhancers’.

We have previously demonstrated that the weakly-cationic small quinolone derived compound HT61 can function as an ‘enhancer compound’ towards AMR pathogens. HT61 has demonstrated activity against Gram-positive *Staphylococcus aureus,* and can also synergise with the aminoglycosides, neomycin and gentamicin, in both planktonic and biofilm conditions [13–15]. Its activity relates to non-specific targeting of anionic lipids within the bacterial membrane leading to rapid partitioning of the lipid bilayer and catastrophic membrane damage [16, 17]. Whilst HT61 has no direct antimicrobial activity against Gram-negative bacteria such as *P. aeruginosa* [14, 15], our group recently reported that when combined with tobramycin, combining HT61 with tobramycin significantly potentiated the bactericidal activity compared to monotherapies in vitro and in vivo [18]*.* Whilst this demonstrated the potential of this therapeutic combination, this research was restricted to a small panel of reference strains of *P. aeruginosa.* This study aimed to further investigate the potential benefit of HT61-tobramycin combinations against 63 clinical isolates of *P. aeruginosa* from sputum and cough samples of patients with CF, using a panel of in vitro assays that assess antimicrobial activity and interactions between the two compounds in both planktonic and biofilm cultures.

## Materials and Methods

### Bacterial Culture Conditions and Maintenance

CF isolates of *P. aeruginosa* were provided by Professor Jane Davies under a material transfer agreement from the CF Bacterial Repository at the National Heart and Lung Institute, Imperial College London. This is a collection of bacteria isolated from airway samples of people with CF at the Royal Brompton Hospital, London. Isolates were stored at -80°C in cryovials as part of the CF Trust-funded PAPA Strategic Research Centre. Cryobeads of isolates were frozen at -80°C in Glycerol-Tryptone Soy Broth (TSB). For all susceptibility assays, Cation-Adjusted Mueller Hinton (CAMH) broth was used [18].

### Drug Preparation

HT61 was provided by Professor Sir Anthony Coates of Helperby Therapeutics and dissolved in 10mg/mL DMSO. Stock solutions were prepared in distilled H_2_O (dH_2_O) at 2.4mg/mL prior to filter sterilisation (0.22µm). Tobramycin stocks were prepared in dH_2_O at 10 or 50mg/mL and filter sterilised (0.22µm). All solutions were stored at -20°C.

### Minimum Inhibitory Concentration (MIC) Assay

HT61 and tobramycin MICs were calculated for each isolate. Modal MICs were determined following EUCAST methodology [18]. Briefly, serial twofold dilutions of tobramycin and HT61 were prepared in CAMH broth in flat-well polycarbonate 96 well plates and treated with 5 × 10^5^ cfu/100 µl. Plates were sealed with a breathable membrane and incubated in a humidified chamber at 37 °C for 16–20 h. MIC experiments were determined using the metabolic dye resazurin to overcome background precipitation [18].

### Chequerboard Assay of Synergy

HT61-Tobramycin combinations were prepared in 96-well plates starting at 2 × MIC followed by serial twofold dilutions in sterile flat well polycarbonate plates creating a total of 64 different combinations. The experiment used the MIC methodology as described above. Fractional inhibitory concentration indices (FICI) were calculated using the concentrations at the growth/no growth interface. FICIs were calculated using the equation:

$$\begin{aligned} & \Sigma {\text{FICA}} + {\text{B}} = {\text{FIC}}_{{\text{A}}} + {\text{FIC}}_{{\text{B}}} \\ & {\text{where}}\;{\text{FIC}}_{{\text{A}}} = {\text{MIC}}_{{{\text{A}} + {\text{B}}}} /{\text{MIC}}_{{\text{A}}} \\ & {\text{and}}\;{\text{FIC}}_{{\text{B}}} = {\text{MIC}}_{{{\text{A}} + {\text{B}}}} /{\text{MIC}}_{{\text{B}}} \\ \end{aligned}$$

The Loewe additivity theory was used with a lower cut-off to determine synergy [19, 20].

### Bacterial Time-Kill Assays

Clinical isolates at 5 × 10^5^ cfu/mL were cultured in the presence of vehicle, tobramycin, HT61, or HT61-tobramycin combinations at sub-inhibitory concentrations identified from preliminary experiments. Bacterial viability was determined by aliquot collection 0, 4, 7, 10 and 24 h post inoculation. Samples of bacterial suspensions were serially diluted in sterile PBS and plated on tryptic soy agar (TSA) for bacterial enumeration.

### Biofilm Eradication Assays

150 µL of bacterial suspensions (1 × 10^5^ cfu/mL) in CAMH were added to a 96-well plate. Plate lids were replaced with Nunc-TSP 96-Pin Lids and biofilms left to establish for 18–20 h at 37 °C under shaking conditions (110 rpm) in a humidified chamber. Established biofilms on pins were then dip-washed with sterile PBS twice and transferred into a challenge plate containing serial dilutions of tobramycin supplemented with HT61 at 25, 50 and 100 µg/mL. For each plate, control wells of tobramycin and HT61 monotherapies were present alongside sterility controls. Biofilms were challenged for 18–20 h under the same conditions described previously and then transferred to antimicrobial-free CAMH. Biofilms were detached from pin lids through high-intensity sonication for 30 min, and recovery plates containing detached biofilms incubated for 24–48 h and used to determine modal minimum biofilm eradication concentrations (MBEC), where modal MBEC was defined as the lowest concentration capable of inhibiting bacterial growth [18].

### Data Presentation and Analysis

MIC values are presented as modal values or range of experimental replicates. Chequerboard analysis was used to identify FICI for different combinations, where synergy was determined when FICI was ≤ 0.5; Additivity when FICI was > 0.5 but ≤ 1.0; no interaction was defined as when the FICI was > 1.0, and antagonist interactions were defined as when the FICI exceeded 4.0 [21]. In vitro time-kill analyses of data are presented as log mean cfu/mL ± standard error of the mean (SEM) and synergy was determined > 2-log reduction in recovered cfu/mL between the combination and monotherapies [22]. Secondary analysis of synergy in bacterial time-kill assays was performed using the BLUR model of synergy [23]. MBEC are expressed as the modal value of experimental replicates and synergy defined as ≥ 4-fold reduction in modal MBEC [24].

## Results

### Differences in MIC Values for Tobramycin Across Different *P. aeruginosa* CF Isolates

MICs for tobramycin and HT61 against 63 different clinical isolates of *P. aeruginosa* are shown in Table 1**.** MICs for tobramycin ranged from 0.13 µg/mL to > 2048 µg/mL. Based on a clinical breakpoint for tobramycin of 2 µg/mL [25], 38 (60%) isolates were classified as tobramycin-resistant, and 25 (40%) isolates were classified as tobramycin-sensitive. In-line with previously work all clinical isolates showed limited susceptibility to HT61, with MICs ranging between 16 and 256 µg/mL.

**Table 1:** Results of susceptibility testing (MICs) for tobramycin and HT61, and the synergy testing (FICIs) of combination therapies for strains 1–63

Isolate ID	Tobramycin Classification	Monotherapy MIC (μg/ml)		Combination MIC (μg/ml)		FICI
		Tobramycin	HT61	Tobramycin	HT61	
1	Resistant	32.00 ± 0.00	128.00 ± 0.00	2.00 ± 0.00	16.0 ± 0.00	0.19 ± 0.00
2	Resistant	13.33 ± 1.69	128.00 ± 0.00	1.33 ± 0.21	16.00 ± 0.00	0.25 ± 0.04
3	Resistant	42.67 ± 6.75	64.00 ± 0.00	4.67 ± 1.12	10.67 ± 1.69	0.27 ± 0.03
4	Resistant	106.67 ± 13.49	170.67 ± 26.98	18.00 ± 4.82	26.67 ± 3.37	0.32 ± 0.03
5	Resistant	2048.00 ± 0.00	256.00 ± 0.00	426.67 ± 53.97	26.67 ± 11.81	0.34 ± 0.05
6	Resistant	32.00 ± 0.00	85.33 ± 13.49	3.67 ± 0.33	18.67 ± 2.67	0.36 ± 0.05
7	Resistant	8.00 ± 0.00	128.00 ± 0.00	2.00 ± 0.45	16.00 ± 0.00	0.38 ± 0.06
8	Resistant	4.00 ± 0.00	32.0 ± 0.00	0.67 ± 0.11	6.67 ± 0.84	0.38 ± 0.05
9	Resistant	16.00 ± 0.00	128.00 ± 0.00	2.00 ± 0.00	32.00 ± 0.00	0.38 ± 0.00
10	Resistant	13.33 ± 1.69	128.00 ± 0.00	2.17 ± 0.60	32.00 ± 0.00	0.42 ± 0.04
11	Sensitive	0.73 ± 0.17	74.67 ± 10.67	0.13 ± 0.04	16.00 ± 0.00	0.43 ± 0.03
12	Sensitive	0.65 ± 0.10	115.20 ± 8.53	0.15 ± 0.03	22.40 ± 2.61	0.45 ± 0.06
13	Resistant	208.00 ± 23.42	104.00 ± 11.71	49.60 ± 6.06	19.20 ± 2.39	0.45 ± 0.05
14	Sensitive	0.25 ± 0.00	102.40 ± 10.45	0.08 ± 0.01	18.40 ± 3.92	0.46 ± 0.08
15	Sensitive	0.44 ± 0.04	48.00 ± 6.05	0.06 ± 0.01	14.00 ± 1.31	0.47 ± 0.06
16	Resistant	26.67 ± 3.37	74.67 ± 17.85	4.67 ± 0.67	12.00 ± 2.53	0.47 ± 0.14
17	Resistant	8.00 ± 0.00	128.00 ± 0.00	2.33 ± 0.56	32.00 ± 0.00	0.5 ± 0.08
18	Resistant	3.33 ± 0.42	128.00 ± 0.00	0.83 ± 0.11	32.00 ± 0.00	0.5 ± 0.00
19	Resistant	16.0 ± 0.00	149.33 ± 35.70	4.50 ± 1.20	32.00 ± 0.00	0.56 ± 0.14
20	Resistant	3.60 ± 0.27	54.40 ± 12.67	0.90 ± 0.07	16.00 ± 2.92	0.58 ± 0.04
21	Resistant	16.0 ± 0.00	51.20 ± 5.23	4.00 ± 0.73	15.20 ± 0.80	0.59 ± 0.08
22	Sensitive	1.50 ± 0.32	48.00 ± 7.16	0.50 ± 0.11	12.00 ± 1.79	0.63 ± 0.06
23	Resistant	20.80 ± 2.44	70.40 ± 10.45	5.60 ± 0.65	19.20 ± 2.13	0.63 ± 0.07
24	Resistant	115.20 ± 8.53	115.20 ± 8.53	35.20 ± 5.23	32.00 ± 0.00	0.63 ± 0.05
25	Sensitive	1.75 ± 0.16	104.00 ± 11.71	0.81 ± 0.26	22.00 ± 3.93	0.64 ± 0.11
26	Sensitive	0.93 ± 0.21	128.00 ± 0.00	0.29 ± 0.06	38.40 ± 4.27	0.64 ± 0.07
27	Sensitive	0.17 ± 0.03	85.33 ± 13.49	0.08 ± 0.02	16.00 ± 0.00	0.67 ± 0.04
28	Sensitive	0.53 ± 0.12	128.00 ± 0.00	0.14 ± 0.03	44.0 ± 5.86	0.67 ± 0.05
29	Resistant	256.00 ± 0.00	64.00 ± 0.00	76.80 ± 8.53	24.00 ± 2.67	0.67 ± 0.04
30	Resistant	1024.00 ± 0.00	128.00 ± 0.00	298.67 ± 42.67	48.00 ± 7.16	0.67 ± 0.05
31	Sensitive	0.38 ± 0.08	48.00 ± 7.16	0.14 ± 0.03	17.33 ± 3.21	0.71 ± 0.10
32	Sensitive	0.50 ± 0.0	42.67 ± 6.75	0.13 ± 0.00	18.67 ± 2.67	0.71 ± 0.04
33	Resistant	> 1024	42.67 ± 6.75	1024.00 ± 0.00	13.33 ± 1.69	0.71 ± 0.11
34	Resistant	6.67 ± 0.84	32.0 ± 0.00	2.00 ± 0.00	13.33 ± 1.69	0.75 ± 0.09
35	Resistant	16.00 ± 0.00	96.00 ± 20.24	5.33 ± 0.84	37.33 ± 8.92	0.75 ± 0.00
36	Resistant	16.00 ± 0.00	42.67 ± 6.75	5.33 ± 0.84	16.00 ± 0.00	0.75 ± 0.09
37	Resistant	512.00 ± 0.00	64.00 ± 0.00	128.00 ± 0.00	32.00 ± 0.00	0.75 ± 0.00
38	Resistant	106.67 ± 13.49	213.33 ± 26.98	32.00 ± 0.00	85.33 ± 13.49	0.75 ± 0.09
39	Resistant	213.33 ± 26.98	53.33 ± 6.75	74.67 ± 17.85	22.67 ± 4.34	0.77 ± 0.08
40	Sensitive	0.75 ± 0.11	128.00 ± 0.00	0.29 ± 0.04	48.00 ± 7.16	0.79 ± 0.04
41	Resistant	5.60 ± 0.65	108.80 ± 9.78	2.30 ± 0.30	36.80 ± 4.80	0.79 ± 0.06
42	Sensitive	0.50 ± 0.00	42.67 ± 6.75	0.21 ± 0.06	18.67 ± 2.67	0.88 ± 0.13
43	Resistant	18.00 ± 3.30	112.00 ± 10.47	6.0 ± 0.76	44.00 ± 7.86	0.88 ± 0.16
44	Sensitive	0.31 ± 0.06	44.80 ± 5.23	0.11 ± 0.01	16.80 ± 3.47	0.91 ± 0.19
45	Sensitive	0.58 ± 0.14	32.00 ± 0.00	0.28 ± 0.08	14.67 ± 1.33	0.92 ± 0.05
46	Sensitive	1.50 ± 0.22	64.00 ± 0.00	1.00 ± 0.32	24.00 ± 5.06	1.00 ± 0.06
47	Resistant	2.33 ± 0.56	64.00 ± 0.00	1.00 ± 0.22	32.00 ± 7.16	1.00 ± 0.22
48	Sensitive	0.42 ± 0.12	106.67 ± 13.49	0.33 ± 0.15	37.33 ± 5.33	1.04 ± 0.12
49	Resistant	2.33 ± 0.56	69.33 ± 12.84	1.25 ± 0.25	29.33 ± 7.64	1.08 ± 0.20
50	Resistant	> 2048	22.40 ± 2.61	960.00 ± 160.46	10.40 ± 1.22	1.08 ± 0.18
51	Sensitive	0.5 ± 0.00	48.00 ± 7.16	0.29 ± 0.04	24.00 ± 3.58	1.13 ± 0.18
52	Resistant	213.33 ± 26.98	64.00 ± 0.00	117.33 ± 10.67	34.67 ± 6.42	1.13 ± 0.05
53	Sensitive	0.29 ± 0.04	128.00 ± 0.00	0.21 ± 0.03	64.00 ± 0.00	1.25 ± 0.11
54	Sensitive	0.42 ± 0.05	128.00 ± 0.00	0.25 ± 0.00	80.00 ± 16.00	1.29 ± 0.23
55	Sensitive	0.25 ± 0.00	16.00 ± 0.00	0.25 ± 0.00	8.00 ± 0.00	1.50 ± 0.00
56	Sensitive	0.58 ± 0.08	53.33 ± 6.75	0.42 ± 0.05	37.33 ± 5.33	1.50 ± 0.18
57	Resistant	10.67 ± 1.69	21.33 ± 3.37	8.00 ± 0.00	13.33 ± 1.69	1.58 ± 0.26
58	Sensitive	0.50 ± 0.00	64.00 ± 0.00	0.42 ± 0.05	53.33 ± 6.75	1.67 ± 0.21
59	Sensitive	1.33 ± 0.21	16.00 ± 0.00	1.13 ± 0.30	16.00 ± 0.00	1.79 ± 0.14
60	Sensitive	0.38 ± 0.08	85.33 ± 13.49	0.36 ± 0.09	80.00 ± 16.00	1.83 ± 0.17
61	Sensitive	1.17 ± 0.17	16.00 ± 0.00	1.00 ± 0.00	16.00 ± 0.00	1.92 ± 0.08
62	Sensitive	0.50 ± 0.00	128.00 ± 0.00	0.46 ± 0.04	128.00 ± 0.00	1.92 ± 0.08
63	Sensitive	1.00 ± 0.00	16.0 ± 0.00	1.00 ± 0.00	16.00 ± 0.00	2.00 ± 0.00

The modal MIC and FICI are given as modal change in MIC (n = 6–10 per strain)

### Combination with HT61 Demonstrates Synergism by Increasing *P. aeruginosa* Sensitivity to Tobramycin

Combination therapy across all clinical isolates using the chequerboard assay of synergy (Table 1) revealed that 47 isolates (isolates 1–47) (74.60%) demonstrated positive interactions (FICI ≤ 1.0) between HT61 and tobramycin**.** Of these 47 isolates, 18 (28.57%, Isolates 1–18) demonstrated synergistic interactions. Only 16 (25.40%) isolates (isolates 48–63) failed to show any positive interactions; no strains demonstrated any evidence of antagonism as determined by a FICI > 4.0. Of the 47 isolates that demonstrated positive interactions, 34 were classified as tobramycin-resistant and 13 classified as tobramycin-sensitive. Combination treatment with HT61 and tobramycin resulted in the conversion of the sensitivity classification for 26.47% (9/34) of isolates from tobramycin-resistant to tobramycin-sensitive.

### Bacterial Time-Kill Analysis Reveals Synergy Between HT61 and Tobramycin Against CF *P. aeruginosa* Isolates

12 of the 63 isolates were selected for further analysis using bacterial time-kill assays. These isolates were selected to include those demonstrating synergy (Isolates 1, 5, 6, 9 and 13), additivity (Isolates 29, 30, 37, 39 and 43) or no interaction (Isolates 61 and 63) based on FICI values. These chosen strains also included strains which demonstrated resensitisation to tobramycin (1, 6 and 9) or remained the same (5, 13, 20, 30, 37, 39, 43, 61 and 63); and covered a range of different changes in MICs (ΔMIC) for tobramycin when used in combination (0–1621 µg/mL).

**Fig. 1:**
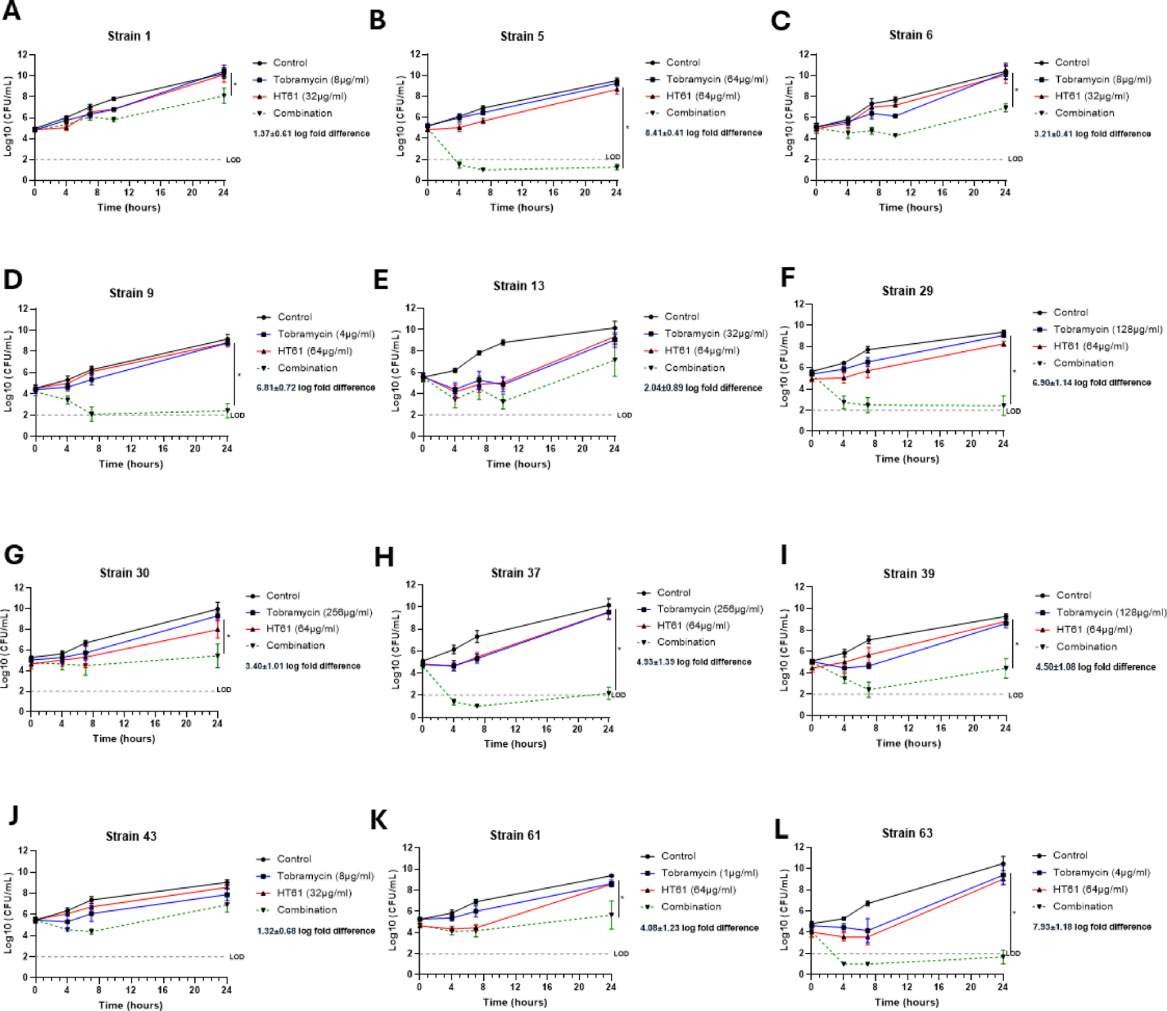
Bacterial time-kill assay demonstrating synergy between tobramycin and HT61 against *P. aeruginosa.* Bacterial time-kill curves for vehicle control (black), sub-inhibitory monotherapy of tobramycin (blue), HT61 (red) and combination (green) against the *P. aeruginosa* CF clinical isolates classified as synergistic (A-E) (**A**), 5 (**B**), 6 (**C**), 9 (**D**), 13 (**E**); isolates classified as additive: 29 (**F**), 30 (**G**), 37 (**H**), 39 (**I**), 43 (**J**) and those with no-interaction: 61 (**K**), 63 (**L**). Samples were collected for enumeration at 0, 4, 7 and 24 h post-treatment. Synergy was determined when combination therapy was > 2 log-fold difference compared to the lowest monotherapy at 24 h. * = P < 0.05 from analysis using the BLUR model of synergy. Data are expressed as mean ± SEM log cfu/mL, N = 4–6 per group. The limit of detection (LOD, 100 cfu/mL) is demonstrated by the grey dotted line

Figure 1 shows time-kill data obtained for this sample set. Preliminary titration experiments were performed for each isolate to identify sub-inhibitory concentrations of HT61 and tobramycin against each isolate. Synergy was accepted as a greater than 2 log-fold difference in bacterial count between combination and individual therapy at 24 h [22]. 3/5 isolates (Fig. 1B–D) demonstrating synergism in the chequerboard assay (FICI ≤ 0.5) also demonstrated synergy in bacterial time-kill assays with a log-order reduction of 6.64 ± 0.41 cfu/mL, 3.05 ± 0.70 cfu/mL, and 5.78 ± 0.47 cfu/mL at 24 h for isolates 5, 6, and 9, respectively. Notable differences between the activity profile of tobramycin when used in combination were observed in these isolates, with isolates 5 and 9 showing a > 3 log cfu reduction within the first 7 h with bacterial counts remaining below the assay’s limit of detection (LOD) for the remainder of the study, indicating bacterial eradication. Conversely, following combination therapy, isolate 6 demonstrated limited efficacy with only a ~ 1 log reduction observed in the first 7 h followed by bacterial regrowth. Neither isolates 1 (Fig. 1A) or 13 (Fig. 1E) reached the threshold for synergy with a log-order reduction of 2.01 ± 0.63 cfu/mL and 1.35 ± 0.88 cfu/mL, but this was within the margin of the calculated SEMs. Secondary analysis using the BLUR model of synergy [23] demonstrated statistically significant synergism (P < 0.05) for isolates 1, 5, 6 and 9 (Table 2).

**Table 2:** Results of BLUR analysis of Synergy for tobramycin-HT61 combinations in bacterial time-kill assays

Strain	TOB % absolute Emax	HT61% absolute Emax	Combination % absolute Emax	BLUR lower limit	BLUR upper limit	Category	Statistical significance
1	20.81	42.43	88.82	49.01	59.41	Synergy	P < 0.05
5	43.69	66.33	100	71.75	81.04	Synergy	P < 0.05
6	31.13	44.74	99.8	55.29	62.17	Synergy	P < 0.05
9	40.36	54.15	100	63.95	72.65	Synergy	P < 0.05
13	59.22	74.31	82.86	79.28	89.52	Additive	Not significant
29	41.19	88.31	99.99	83.29	93.12	Synergy	P < 0.05
30	57.63	71.84	99.52	78.23	88.07	Synergy	P < 0.05
37	70.71	92.33	100	90.38	97.75	Synergy	P < 0.05
39	62.93	51.89	99.95	72.32	82.17	Synergy	P < 0.05
43	58.63	53.03	81.49	72.75	82.64	Additive	Not significant
61	68.33	78.46	98.77	82.47	93.18	Synergy	P < 0.05
63	72.09	67.29	100	81.45	90.87	Synergy	P < 0.05

Secondary analysis using the Bliss-Loewe Uncertainty Range Model (BLUR) [23] was performed on all strains evaluated in the bacterial time-kill assays

Synergy was detected in 4 out of 5 (Fig. 1F–I) isolates defined as additive by the chequerboard assay, with a log-order reduction of 5.13 ± 0.82 cfu/mL, 2.27 ± 0.32 cfu/mL, 6.36 ± 0.71 cfu/mL, and 3.84 ± 0.84 cfu/mL at 24 h for isolates 29, 30, 37, and 39, respectively. Notable differences were again observed in the killing profile of tobramycin when used in combination. Isolates 29, 37 and 39 demonstrated a ≥ 3 log-fold reduction within the first 7 h which remained below the LOD for the remainder of the assay, with the exception of 37 which demonstrated modest bacterial regrowth 24 h post treatment. Isolate 30 demonstrated stable bacterial numbers throughout the duration of the experiment with < 1 log reduction suggestive of a bacteriostatic response. BLUR analysis confirmed statistically significant synergism (P < 0.05) for isolates 29, 30, 37 and 39 (Table 2). Only strain 43 (Fig. 1J) failed to show synergy in the time-kill assay with a log-order reduction of 1.80 ± 0.77 cfu/mL observed following 24 h of treatment. However, this remained within the margin of the calculated SEMs.

Isolates 61 and 63 which showed no interaction based on FICI values demonstrated synergistic bactericidal activity with log-order reductions of 3.39 ± 0.97 and 6.13 ± 0.82 for 61 and 63, respectively (Fig. 1K, L). BLUR analysis also confirmed statistically significant synergism (P < 0.05) for both isolates (Table 2).

### HT61 Significantly Enhances the Bactericidal Activity of Tobramycin in Established Biofilms of CF *P. aeruginosa* Isolates

**Fig. 2:**
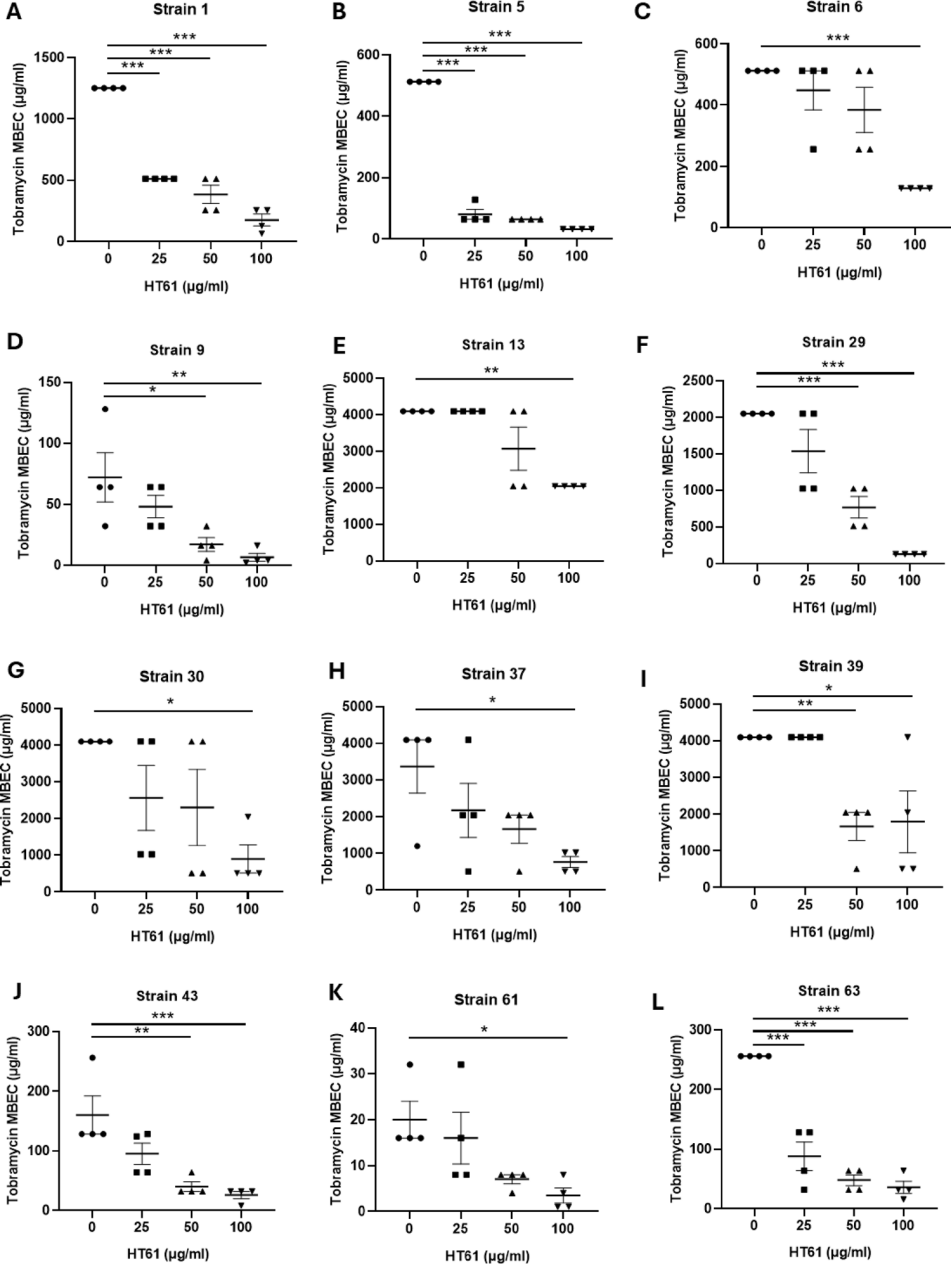
HT61 reduces the concentration of tobramycin required to eradicate *P. aeruginosa* in established biofilms*.* Increasing concentrations of tobramycin were combined with fixed concentrations of HT61 (0, 25, 50 or 100 µg/mL) and used to challenge 24 h old established biofilms of the *P. aeruginosa* CF clinical isolates classified as synergistic: 1 (**A**), 5 (**B**), 6 (**C**), 9 (**D**), 13 (**E**); isolates classified as additive: 29 (**F**), 30 (**G**), 37 (**H**), 39 (**I**), 43 (**J**) and those with no-interaction: 61 (**K**), 63 (**L**). The MBEC was determined as the lowest concentration capable of eradicating visible bacteria within a biofilm following antibacterial challenge of established biofilms. Data are expressed as modal tobramycin MBEC (n = 4 per group). Synergy was accepted when a combination therapy produced fourfold or greater reduction in modal MBEC. * = P < 0.05, ** P < 0.01, *** P < 0.001 compared to vehicle control following a one-way ANOVA and Dunnett’s multiple comparison test

Synergy was demonstrated with the combination of tobramycin and HT61 against Isolates 1, 5, 6, 9 in the chequerboard assay and against biofilms, as defined by a ≥ 4-fold reduction in modal MBEC between tobramycin alone or in combination [24] (0 vs 100 µg/mL HT61, P < 0.01). Isolates 1 and 6 (Fig. 2A and C) demonstrated a 4-fold reduction, and isolates 5 and 9 (Fig. 2B and D) demonstrated a 16-fold reduction when treated with tobramycin combined with 100 µg/mL HT61 as compared to tobramycin alone. Whilst all 5 strains demonstrated a statistically significant concentration-dependent reductions in MBEC when treated with tobramycin, the reduction in MBEC for Isolate 13 (Fig. 2E) did not meet the criteria for synergy in this biofilm assay as only a twofold reduction in modal MBEC was seen with 100 µg/mL HT61.

The combination of tobramycin and HT61 demonstrated additive interactions against Isolates 29, 30, 37, 39 and 43 based on FICI values but demonstrated synergy against biofilm cultures with 16-fold, 8-fold, 4-fold, 8-fold and 4-fold reductions (Fig. 2F–J, P < 0.05) respectively. Furthermore, a significant concentration-dependent reduction in MBEC was seen for all isolates tested, in agreement with the time-kill analyses except for isolate 43, which did not meet the threshold for synergism suggesting greater activity of the combination against biofilm resident *P. aeruginosa*.

The combination of tobramycin and HT61 also demonstrated synergy against biofilms of Isolates 61 and 63 (Fig. 2K, L) with fold reductions of 16 and 8, respectively, again demonstrating a significant concentration-dependent reduction in MBEC despite showing no interactions based on FICI values.

## Discussion

Lower respiratory tract infections are one of the leading causes of death globally. With the rise in antibiotic resistance, effective treatment remains a significant unmet clinical need. Here, we demonstrate that HT61-tobramycin combinations demonstrate antimicrobial synergy in 63 CF clinical isolates of *P. aeruginosa*, expanding on previous work [13, 18, 26]. Chequerboard analysis revealed that 74% of isolates demonstrated either positive interactions (29/63) or synergy (18/63) as determined by FICI values of below 1.0. Furthermore, when a selection of these isolates covering all interaction categories were assessed (synergistic, positive or indifferent interactions), tobramycin activity was clearly augmented in planktonic cultures. Crucially, this activity was replicated against biofilm cultures highlighting the combinations potential therapeutic capabilities against bacteria in different life phases relevant to *P. aeruginosa* infections in CF and COPD patients.

Historically, methods for overcoming AMR in pulmonary infections with *P. aeruginosa* has focused around increased antibiotic dosages. However, for antibiotics such as tobramycin which already exhibits significant systemic toxicity profiles including oto- and nephro-toxicity, increasing the antibiotic dose further is unfeasible. Therefore, alongside our previous work [18], these findings highlight the ability of HT61 to reduce the required concentrations of tobramycin needed for antimicrobial activity, thereby potentially reducing systemic toxicity associated with long-term tobramycin therapy in chronic respiratory infections. The extent to which HT61 augments tobramycin activity against both drug-sensitive and -resistant profiles is striking. Whilst 30% of tobramycin-resistant isolates demonstrated a re-sensitisation of their susceptibility to the aminoglycoside (conversion of MICs from > 2 µg/mL to < 2 µg/mL) when treated with the combination, 68% (26/38) of tobramycin resistant isolates demonstrated a minimum of a 4-fold change in MIC.

Through its cationic structure, HT61 targets anionic lipids such as phosphatidylglycerol and phosphatidylethanolamine within the bacterial membrane leading to impaired membrane integrity and catastrophic membrane damage [15, 27, 28]. We therefore hypothesise that HT61 augmentation of tobramycin activity is achieved through membrane disruption and increased intracellular antibiotic penetration. This is supported by other positively charged antibiotics including Polymyxin B and E, which also induce membrane disruption through displacement of divalent cations demonstrating synergy with a number of conventional antibiotics, including aminoglycosides and carbapenems, against MDR Gram-negative pathogens *P. aeruginosa* and *K. pneumoniae* [29, 30]. Future work quantifying intracellular antibiotic penetration using dye incorporation assays, such as 1-*N*-phenylnapthylamine (NPM), could be utilised to validate this hypothesis.

Whilst the potentiation of tobramycin activity by HT61 was shown across most *P. aeruginosa* isolates, the antimicrobial activity profiles varied with both bactericidal and bacteriostatic profiles observed. Antimicrobial resistance of Gram-negative pathogens, including *P. aeruginosa,* to aminoglycosides such as tobramycin may develop through several distinct mechanisms including (1) increased antibiotic extrusion through increased efflux pump expression, (2) enzymatic modifications that inactivate the antibiotic, (3) decreased membrane permeability to antibiotics through reduced porin expression, and (4) modifications to its target, the ribosomal 30S subunits in *P. aeruginosa* [31]*.* Based on our hypothesis of HT61 synergism with tobramycin, the specific mechanism of antimicrobial resistance development could have significant implications on the combination’s activity. For example, isolates with resistance mechanisms associated with reduced membrane permeabilization may be more responsive to HT61-tobramycin combinations through HT61-mediated membrane disruption and increased tobramycin entry. In support of this, extensive synergy has been reported between known membrane permeabilisers and antibiotics that are otherwise inactive against Gram-negative bacteria such as polymyxin B nonapeptide and rifampicin recently reviewed by Wesseling and Martin [32]. In contrast, the beneficial effects of HT61-tobramycin combinations may be offset by resistance mutations associated with increased efflux pump activity reducing intracellular exposure, such as increased expression of the MexXY efflux pump in *P. aeruginosa*. It is important to note that strains may possess complex resistance phenotypes which are not restricted to isolated mechanisms of resistance, and the mechanism of tobramycin resistance being only one factor influencing the observed potentiation. Therefore, alongside genotyping of the strains used in this study, additional studies using isogenic strains with known mutations would allow for a definitive assessment of how resistance phenotypes impact the antimicrobial activity of HT61-tobramycin combinations, not only in *P. aeruginosa,* but also in other bacterial species.

In conclusion, this study highlights a significant therapeutic potential of HT61-tobramycin combination therapy for the treatment of chronic respiratory infections with MDR pathogens. The synergistic combination of such agents could allow significant reduction in antibiotic doses required to treat such infections, specifically mitigating adverse symptoms such as ototoxicity and nephrotoxicity [33–35], highlighted as a key deliverable for people with CF by the recent James Lind Alliance priority setting exercise [36]. This study therefore demonstrates the further characterisation of such combinations as a novel strategy against a range of respiratory illnesses in the fight against antimicrobial resistance.

## Data Availability

The data presented in this study are available on request from the corresponding authors.
